# Ewing's Sarcoma of Ilium, Presenting as Right Lower Quadrant Pain

**DOI:** 10.1155/2015/629601

**Published:** 2015-11-30

**Authors:** Osama Saleh Alshaya, Munzir Izzeldin Abbasher, Mubashir Maqbool Wani

**Affiliations:** Department of Orthopedics, Division of Surgical Specialities, King Fahad Medical City, Riyadh 11525, Saudi Arabia

## Abstract

Ewing's sarcoma is a highly malignant tumor of bone and is more common in children in the age group of 10 to 20 years. Sometimes the classic clinical and radiological presentation of Ewing's sarcoma may not be the norm and patient may have an atypical presentation leading to diagnostic confusion. This situation is especially true for Ewing's sarcoma involving iliac bone. We report a case of Ewing's sarcoma involving the right ilium in a patient presenting with right lower quadrant pain and nonspecific radiological changes. To the best of our knowledge, this scenario has not been reported in literature. We recommend early magnetic resonance imaging and computed tomography to diagnose the disease early when there is slightest suspicion of the disease.

## 1. Introduction

Ewing's sarcoma is known for being highly malignant. Named after James Ewing [1], it is the second most common primary malignant tumor of bone in children with a characteristic predilection for an age group between 10 and 20 years [2]. Although the exact etiology is not known, it has been found that, in 85% of ES patients, a characteristic t(11;22) chromosomal translocation is found. The location of ES is most often in the pelvis and lower extremity [3]. In 12.5% cases of ES, iliac bone is the site of origin. Clinically, the most common presenting symptom is pain (90%), followed by swelling (70%) [4]. Radiologically, it is described as a central, diaphyseal, lytic tumor, which is often permeative and has a lamellated or “onion skin” periosteal reaction when it affects a long bone often associated with soft tissue mass. The bone lesions are usually lytic but may be sclerotic or mixed. Most of the lesions are diaphyseal or metadiaphyseal. Diagnosing Ewing's sarcoma of ilium remains a challenge in its early stages. This is partly because of the myriad of symptoms with which it presents and the very subtle changes radiographically when in its initial stage. We describe one such case in a patient, who had Ewing's sarcoma of right ilium and presented with pain in the right lower quadrant of the abdomen. We also reviewed the literature regarding the diagnostic dilemma of Ewing's sarcoma of ilium and the different clinical presentations it can have.

## 2. Case Report

**Figure 1:**
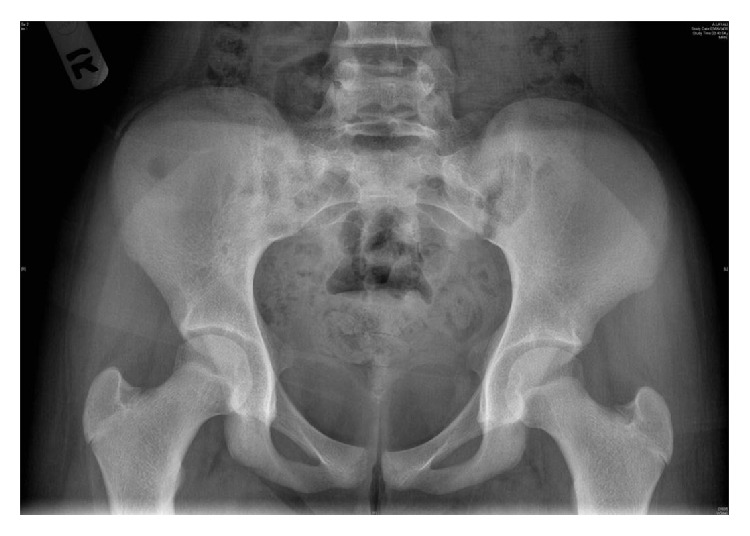
X-ray AP view of the pelvis showing hard to pick changes in the right ilium.

**Figure 2:**
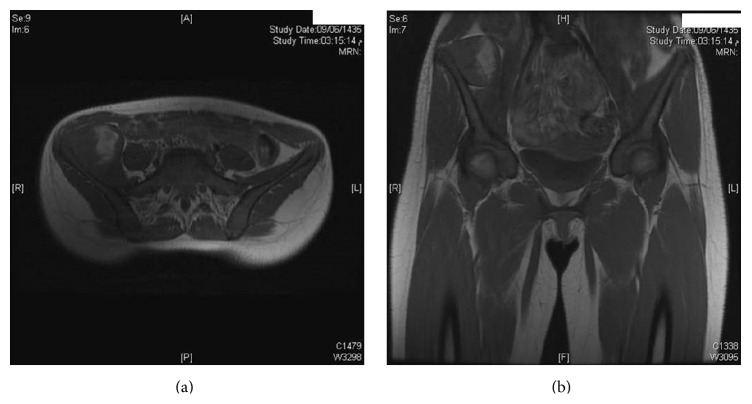
MRI images of the patient before initiation of neoadjuvant chemotherapy, showing mass arising along the right iliac bone, most probably soft tissue sarcoma with focal bony involvement.

**Figure 3:**
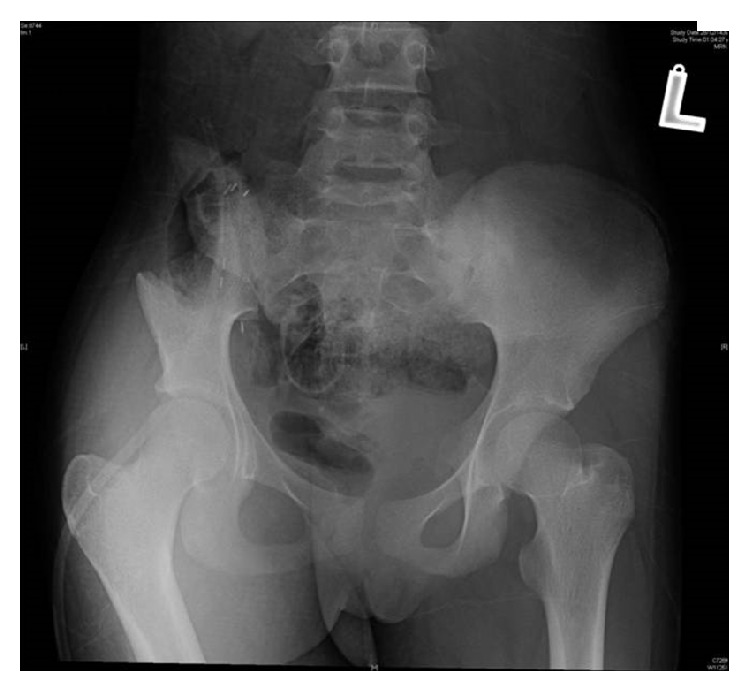
X-ray image of the patient showing right internal hemipelvectomy.

**Figure 4:**
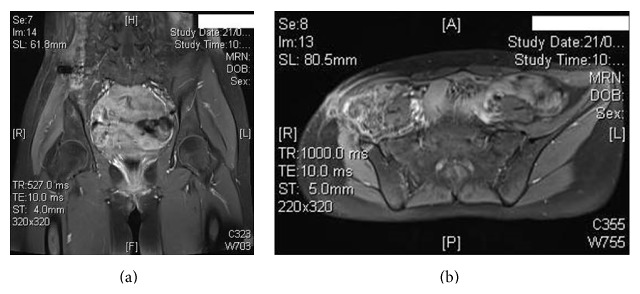
MRI images of the patient showing no evidence of tumor.

A girl, 13 years of age, Saudi in origin, presented to us with a history of pain in the right lower quadrant of 6-month duration. She had visited many clinics for her pain with no relief to her symptoms. In the initial stages of her disease, she had undergone multiple ultrasound examinations with no clue of the diagnosis. The patient already had undergone appendectomy 2 years back, so appendicitis was not kept as a differential diagnosis. In one of the latest ultrasound examinations of the abdomen, the sonologist picked up a mass in her right iliac fossa arising from pelvis suspecting an abscess or a tumor. The patient was referred to our clinic for further evaluation and treatment. On our examination, there was no tenderness in the right iliac fossa nor could any swelling be palpated. ESR, CBC, and CRP were normal. X-ray of the pelvis (AP) was done and did not show any significant bony changes (Figure 1). MRI of the pelvis was done which showed a soft tissue mass arising along the right iliac bone, most probably soft tissue sarcoma with focal bony involvement (Figures 2(a) and 2(b)). This was followed by technetium-99m MDP scintigraphy. This study was negative for multifocal skeletal metastatic disease and negative for local invasion of the osseous structures. CT scan of thorax and abdomen was done which ruled out any metastasis. Under sterile conditions, ultrasound-guided fine needle aspiration of the right iliac fossa mass was done using 22 G needle. About 15 mL hemorrhagic fluid was aspirated. This showed presence of malignant cells. It was followed by an open biopsy of the mass which was reported by the histopathologists as Ewing's sarcoma. Patient was put on neoadjuvant chemotherapy which included vincristine, cyclophosphamide, doxorubicin and etoposide, and ifosfamide (VCD/IE 3 times weekly). Following neoadjuvant chemotherapy, the mass had shrunk in size and internal hemipelvectomy was undertaken excising the tumor mass along with the involved bone sparing the sacroiliac joint and hip joint (Figure 3). The margins were negative for any residual tumor. The patient was again given chemotherapy after surgery and radiotherapy was added to minimize the chances of recurrence. Presently, patient is doing fine and is ambulatory and pain-free. The last MRI done for the patient does not show any evidence of recurrence (Figures 4(a) and 4(b)). The patient will follow up with us regularly.

## 3. Discussion

Early diagnosis of Ewing's sarcoma of ilium remains challenging partly because of the subtle difficult to detect changes in the radiographs and the nonspecific clinical symptoms that patients have. After reviewing the literature by searching Pubmed with terms such as “Ewing's sarcoma of ilium,” “Diagnosis of Ewing's sarcoma of ilium,” and “clinical presentations of Ewing's sarcoma,” we could find few case reports regarding the atypical presentation of Ewing's sarcoma. The delay in diagnosis can partly be due to the radiologic appearance of inflammatory and tumorous lesions in the iliac bone which is characterized by destructive alterations and consolidations simultaneously. This pattern is nonspecific. The value of plain films of this area is compromised by the anatomy of the iliac bone and by overlying structures [5]. There have been instances where authors have diagnosed Ewing's sarcoma of the ilium as sacroiliitis [6–8] and in some cases even septic arthritis [9] possibly because of transarticular spread in these patients. The authors of these cases mainly relied on clinical presentation and plain radiography initially which lead to the diagnostic confusion. There have also been cases reported of Ewing's sarcoma of ilium mimicking juvenile rheumatoid arthritis [10] and pain in the hip [11]. In our case the patients presentation as right lower quadrant pain again led to diagnostic confusion and delayed treatment till a definite diagnosis could be reached. Ultrasound examination has been suggested in the past as a tool in evaluating the presence and extent of the extraosseus component as well as assessing the therapeutic response of tumors which originate in the skeleton in close proximity to pelvis [12] but with present day modern diagnostic methods its role is very limited.

Patients of Ewing's sarcoma need a multidisciplinary approach to treatment which include involvement of oncologists, radiation oncologists, surgeons, and radiologists. While surgery is effective and appropriate for patients who can undergo complete resection with acceptable morbidity, children who have unresectable tumors or who would suffer loss of function are treated with chemotherapy and radiation therapy alone. We were fortunate enough to have clear margins at the time of surgery in our patient without causing any functional disability to the patient. Prognosis depends on extent of the disease, size, and location of the tumor, presence or absence of the tumor metastasis, tumor response to therapy, age, and disease relapse. Most centers today report long-term survival of 60% to 70%. The worst prognostic factor is the presence of distant metastasis. Even with aggressive treatment, patients with metastasis have only a chance of 20% long-term survival. Histological grades are of no prognostic significance.

## 4. Conclusion

We believe that Ewing's sarcoma should be kept as one of the rare differential diagnoses for lower quadrant pain. Ultrasonography should not be relied on much and X-rays may not add more to the knowledge. Lower threshold should be kept for investigations such as CT and MRI to detect the disease early and start the appropriate treatment.
